# Same-day Subcutaneous Implantable Cardioverter-defibrillator Placement and Discharge: Another Step Toward Outpatient Electrophysiology

**DOI:** 10.19102/icrm.2020.110606

**Published:** 2020-06-15

**Authors:** Christopher R. Ellis

**Affiliations:** ^1^Cardiovascular Division, Vanderbilt Heart and Vascular Institute, Vanderbilt University Medical Center, Nashville, TN, USA

**Keywords:** Same-day discharge, subcutaneous implantable cardioverter-defibrillator, telemedicine

Subcutaneous implantable cardioverter-defibrillators (ICDs) (S-ICDs) have slowly been gaining a reputation as a cardiac implantable electronic device option that imparts a lower surgical risk, particularly among younger patients requiring ventricular fibrillation therapy or those patients with multiple comorbid illnesses who are at high risk for adverse outcomes if given transvenous systems. Though we have not yet seen a landmark randomized trial of comparison, existing registry and prospective cohort studies do favor^1,2^ S-ICDs over transvenous ICDs when considering the risks for lead fracture, lead dislodgement, pneumothorax, and venous thrombosis. Meanwhile, although the risk of infection (ie, in the device pocket) may be comparable between the two modalities,^1,2^ the risk of endocarditis should be expected to be nearly nonexistent with the S-ICD given the avoidance of vascular access. However, the cost of the S-ICD does come at a small premium and there are also the additional concerns of general anesthesia and hospitalization for postoperative pain control adding to the perceived expenditures, which may act as a barrier to adoption in some centers.

In this issue of *The Journal of Innovations in Cardiac Rhythm Management*, the results of a small series of patients at Riverside Hospital in Columbus, Ohio are reported. Among patients (n = 24) who underwent S-ICD, half of whom were sent home on the same day as device implantation (n = 13), Swinning et al.,^3^ found no specific increased risk. A reasonable health care system cost savings of $1,664.48 per patient was reaped with the protocol of same-day early discharge after S-ICD implantation. In such a study, specific details matter, with patients being excluded if they lived more than 30 miles away from an emergency room, if they did not have a driver who could stay overnight with them at home, or if their procedure was completed after 1:00 pm. Based on a heavy male bias (83% male), one could suspect that sex was also an exclusion or played a role in the perceived pain tolerance and stability of the patient that made them eligible for same-day discharge, although the sample size is too small to draw definitive conclusions of this kind.

In our experience, the most likely issue to appear after S-ICD implantation is local site pain, which can be affected by patient size or body mass index^4^ or by an individual’s pain tolerance, which is driven both by psychological^5^ and physiological^6^ factors. Longer-acting and carefully placed local anesthesia is key to better pain outcomes during the first six to 12 hours postsurgery. Swinning et al. do not mention a specific protocol for adjunctive therapy such as the use of nonsteroidal anti-inflammatory drugs like ketorolac or ibuprofen on a scheduled basis, which could help to alleviate late inflammatory pain effects. Also, it would be useful to assess the impact of interrupted direct oral anticoagulants/novel oral anticoagulants or oral anticoagulation and antiplatelet therapy on the decision to pursue same-day discharge as well as any impact that may occur on the rate of presentation of delayed hematoma requiring evaluation or management.

It is important to note this study was performed before the coronavirus disease 2019 (COVID-19) pandemic and the resultant widespread adoption of telemedicine visits for clinic, consults, and other meetings to avoid risking exposure of both patients and providers alike to the virus.^7^ Still, the authors’ use of telemedicine visits for wound checks and patient assessments as an element of routine care following S-ICD placement or to triage issues that arose in the early postoperative period among same-day discharge patients is to be commended. Both this study and the data we are now gathering from efforts to continue patient care during the ongoing COVID-19 pandemic support the potential for greater telemedicine adoption in medicine, potentially leading to an earlier diagnosis of problems and subsequent reductions in disease severity or complications. More specifically, in the area of cardiac electrophysiology, this study clearly marks another step toward alleviating hospital stay expenses and mobility limitations together with the other research available on same-day discharge after cardiac resynchronization therapy device implantation,^8^ laser lead extraction (personal experience), atrial fibrillation ablation,^9^ and left atrial appendage closure.^10^
